# Supplementary material for: Attribution Markers and Data Mining in Art Authentication
Source: Molecules. 2021 Dec 23;27(1):70. doi: 10.3390/molecules27010070 (PMC8747058; doi:10.3390/molecules27010070)
Supplement: Supplementary file 1 [file molecules-27-00070-s001.zip › molecules-1461782-supplementary.pdf]

Table S1. The list of the analysed paintings

Lp.	Paintings
1	Willem & Joan Blaeu, *Theatrum orbis terrarum sive Atlas Novus*, Amsterdam 1649–1655, Ossoliński National Institute *
2	J.J.Knechtl "*Swięty Augustyn*" (Jawor) ca.1730*
3	J.J Knechtl "*Bolko II Swidnicki*" ca. 1720*
4	M.L.Wilmann "*Chrystus przy kolumnie*" ca. 1701, National Museum in Wrocław*
5	A. Grottger "Ekwipaż" ca. 1849, National Museum in Wrocław, Ossoliński National Institute*
6	Johann Baptist Homann, "*Imperium Romano-Germanicum Germanicum in suos Circulos divisum Geographice exhibitum et in Usum Scholarum designatum* […]", Norymberga 1732*
7	A.Grottger „*Gra w Marasza*" ca. 1858-1960 , Ossoliński National Institute*
8	Sofonisba Anguissola „*Gra w szachy*" MNP*
9	H. Siemiradzki "*Idylla*" private collection**
10	Johann Baptist Homann, „*Statuum totius Italiae novissima repraesentatio geographica simul exhibens Insulas Siciliae Corsicae et Maltae connate*, Norymberga 1729*
11	M.L. Willmann „*Św. Jan Kapistran*", ca. 1693*
12	J.J. Knechtl „*Karol Boromeusz*" Przychowa ca. 1721*
13	A.Grottger „*Krajobraz górski*" ca. 1855 , Ossoliński National Institute*
14	M.L. Willmann „*Męczeństwo sw Barbary*" ca. 1680-85, National Museum in Wrocław*
15	M.L. Willmann „*Koronacja Najświętszej Marii Panny*" 1685, National Museum in Wrocław*
16	J.J Knechtl „*Świety Jan Niepomucen*" Lubomierz ca.1736*
17	J.J. Knechtl „*Świety Jan Niepomucen*" Przychowa ca. 1721*
18	M.L. Willmann „*Orfeusz grający zwierzętom*" 1670, National Museum in Wrocław*
19	G. Penni "*The Holy Family with Saint John and Saint Catherine*" Childs Gallery in Boston (*access to the data from Harvard Institute*)
20	G. Penni "*The Holy Family with Saint John and Saint Catherine*" National Museum in Warsow*
21	A. Grotter „*Po powstaniu*" 1864, National Museum in Wrocław*
22	A. Grotter „*Portret Jerzego Lubomirskiego*" 1866, National Museum in Wrocław*
23	M.L. Willman „ *Porwanie Persefony*" ca 1665 National Museum in Wrocław*
24	Vincenzo Maria Coronelli, *"Silesia Inferiore",* Dedicata Dal P. Cosmografo Coronelli All Illustrissimo et Eccelentissimo S. Gio. Battista Gradenigo, Senatore Amplissimo, Wenecja 1692, Ossoliński National Institute*
25	Abraham Ortelius, „*Silesiae typus",* A Martino Helwigio Nissense descriptus et Nobili docto-que viro Domino Nicolao Rhedingero ded., Antwerp 1603, Ossoliński National Institute*
26	M.L. Willmann „*Świeta Rodzina*" 1678, National Museum in Wrocław *
27	A. Grotter „*Szarża ułanów*" 1855 , Ossoliński National Institute*
28	A. Grotter „*Szpica polska*" ca. 1855, Ossoliński National Institute*
29	J. Tintoretto (1518-1594) „ *Narcyz*" private collection*
30	A. Grotter „*Towarzystwo w parku*" 1864 r, Ossoliński National Institute*
31	J.J. Knechtl „*Ukrzyżowanie*" Nowe Miasteczko 1740~1750*
32	J.J. Knechtl „*Ukrzyżowanie*" Nysa ca.1750*
33	M.L.Willmann „*Wizja Świetego Antoniego Padewskiego*" 1699*
34	Wyspiański "*Szkic do Apolla*" initially atributted to S. Wyspiański**
35	Drawings of the painting "*Chrystus wśród dzieci*" initially atributted to W. Wojtkiewicz**
36	M. Gierymski (1846-1874) *Pastel with fruits***
37	„*Wiosna w małym miasteczku*" initially atributted to M.Gierymski**
38	Norblin „*Scena batalistyczna*" initially atributted to Norblin**
39	"*Portret Kobiety*" initially atributted to Olga Boznańska**
40	Pastel depicting the landscape, initially atributted to S. Wyspiański**
41	„ *Uczniowie z Emaus*" Han van Megeren,data from literature [6]
42	„*Madonna*" initially atributted to Botticelli**
43	„ *Madona z dzieckiem*" initially atributted to Camino**
44	El Greco "*Ekstaza świętego Franciszka*" ca 1575 – 1580, The diocese museum in Siedlce * &**
45	M. Gierymski , Unknown painting, private collection**
46	H. Siemiradzki, Unknown painting, private collection**
47	M. Gierymski "*Jabłoń nad potokiem*" ca. 1867, National Museum in Kraków**
48	H. Siemiradzki "*Pochodnie Nerona*" 1876, National Museum in Kraków*
49	M. Gierymski "*Polowanie par force na jelenia*" 1874, National Museum in Kraków**
50	H. Siemiradzki "*Scena męczeństwa pierwszych chrześcijan*", National Museum in Kraków**
51	M. Gierymski "*Zwiady kozaków kubańskich*" 1868-1869, National Museum in Kraków**
52	"*Sąd Ostateczny*" paiting atributted to H. Bosch, The Royal Castel Wawel in Kraków*&**
53	J.J. Knechtl "*Madonna z dzieckiem*" ca 1739*
54	"*Chrystus w Ogrójcu*" initially attributed to M.L. Willman*
55	„*Madonna z dzieckiem i aniołem*" initially atributted to F. Francia**

*The studies were conducted by the Cultural Heritage Research Laboratory at the University of Wroclaw (Poland)

** The studies were conducted by the Laboratory of Analysis and Non-Destructive Investigation of Heritage Objects (LANBOZ) in Krakow (Poland)
